# A Bi-Functional Anti-Thrombosis Protein Containing Both Direct-Acting Fibrin(ogen)olytic and Plasminogen-Activating Activities

**DOI:** 10.1371/journal.pone.0017519

**Published:** 2011-03-14

**Authors:** Hailong Yang, Yipeng Wang, Yao Xiao, Ying Wang, Jing Wu, Cunbao Liu, Huahu Ye, Fengliang Li, Haining Yu, Ren Lai

**Affiliations:** 1 Biotoxin Units of Key Laboratory of Animal Models and Human Disease Mechanisms, Kunming Institute of Zoology, Chinese Academy of Sciences, Kunming, Yunnan, China; 2 Life Sciences College of Nanjing Agricultural University, Nanjing, Jiangsu, China; 3 School of Life Science and Biotechnology, Dalian University of Technology, Dalian, Liaoning, China; 4 Department of Laboratory Medicine, The First People's Hospital of Yunnan Province, Kunming, Yunnan, China; 5 Institute of Biotechnology, The Academy of Military Medical Sciences, Beijing, China; 6 Graduate School of the Chinese Academy of Sciences, Beijing, China; University of South Florida College of Medicine, United States of America

## Abstract

Direct-acting fibrin(ogen)olytic agents such as plasmin have been proved to contain effective and safety thrombolytic potential. Unfortunately, plasmin is ineffective when administered by the intravenous route because it was neutralized by plasma antiplasmin. Direct-acting fibrin(ogen)olytic agents with resistance against antiplasmin will brighten the prospect of anti-thrombosis. As reported in ‘Compendium of Materia Medica’, the insect of *Eupolyphaga sinensis* Walker has been used as traditional anti-thrombosis medicine without bleeding risk for several hundreds years. Currently, we have identified a fibrin(ogen)olytic protein (Eupolytin1) containing both fibrin(ogen)olytic and plasminogen-activating (PA) activities from the beetle, *E. sinensis*. *Objectives:* To investigate the role of native and recombinant eupolytin1 in fibrin(ogen)olytic and plasminogen-activating processes. *Methods and* *Results*: Using thrombus animal model, eupolytin1 was proved to contain strong and rapid thrombolytic ability and safety *in vivo*, which are better than that of urokinase. Most importantly, no bleeding complications were appeared even the intravenous dose up to 0.12 µmol/kg body weight (3 times of tested dose which could completely lyse experimental thrombi) in rabbits. It is the first report of thrombolytic agents containing both direct-acting fibrin(ogen)olytic and plasminogen-activating activities. *Conclusions:* The study identified novel thrombolytic agent with prospecting clinical potential because of its bi-functional merits containing both plasmin- and PA-like activities and unique pharmacological kinetics *in vivo*.

## Introduction

The incidence of thrombotic disorders including cerebral stroke, myocardial infarction, and venous thromboembolism are rapidly increasing throughout the world. Thrombolytic therapy is an acknowledged approach to treat these disorders. All thrombolytic agents in current clinical usage are plasminogen activators (PA) which require plasminogen to achieve thrombosis. Although effective, PAs uniformly increase the risk of bleeding complications, especially intracranial hemorrhage, and no laboratory test is applicable to avoid such bleeding [1]. This issue arouses the attempt to identify improved regents that would avoid hemorrhage complications and increase thrombolytic potential. Plasmin is a “direct-acting” thrombolytic agent that had been considered to contain greater thrombolytic efficacy and safety than can be afforded by PAs. Although plasmin is direct fibrinolytic enzyme without requirement of plasminogen, it is ineffective when administered by the intravenous route because it was neutralized by plasma antiplasmin [1]–[3].

Some anti-thrombosis components including thrombin inhibitors, platelet aggregation inhibitors, vasodilators, fibrin(ogen)olytic enzymes, apyrases and peroxidases have been identified from some medicinal insects [4]–[11]. Recently, 12 fibrin(ogen)olytic enzymes have been found in the salivary glands of the horsefly, *Tabanus yao* Macquart [8], [9], suggesting that some medical insects contain diverse direct-acting thrombolytic agents. In oriental regions, a number of traditional treatment regimens have been used for thrombotic diseases and many of these crude drugs have been evaluated through the purification and characterization of the active substances from insects [8]–[13]. The ground beetle of *E. sinensis* has been used in East Asia as crude drugs for thrombolytic therapy and is presently reared as a medicinal insect in China. Recently, fibrinolytic activities have been found in this insect but the component was not characterized [14]. In order to identify and characterize the interesting thrombolytic compounds and help in identifying novel thrombolytic candidates, we investigate thrombolytic molecules in the insect of *E. sinensis* in the current work.

## Results

### Identification of anatomical position in the ground beetle with fibrinogenolytic activity

**Figure 1:**
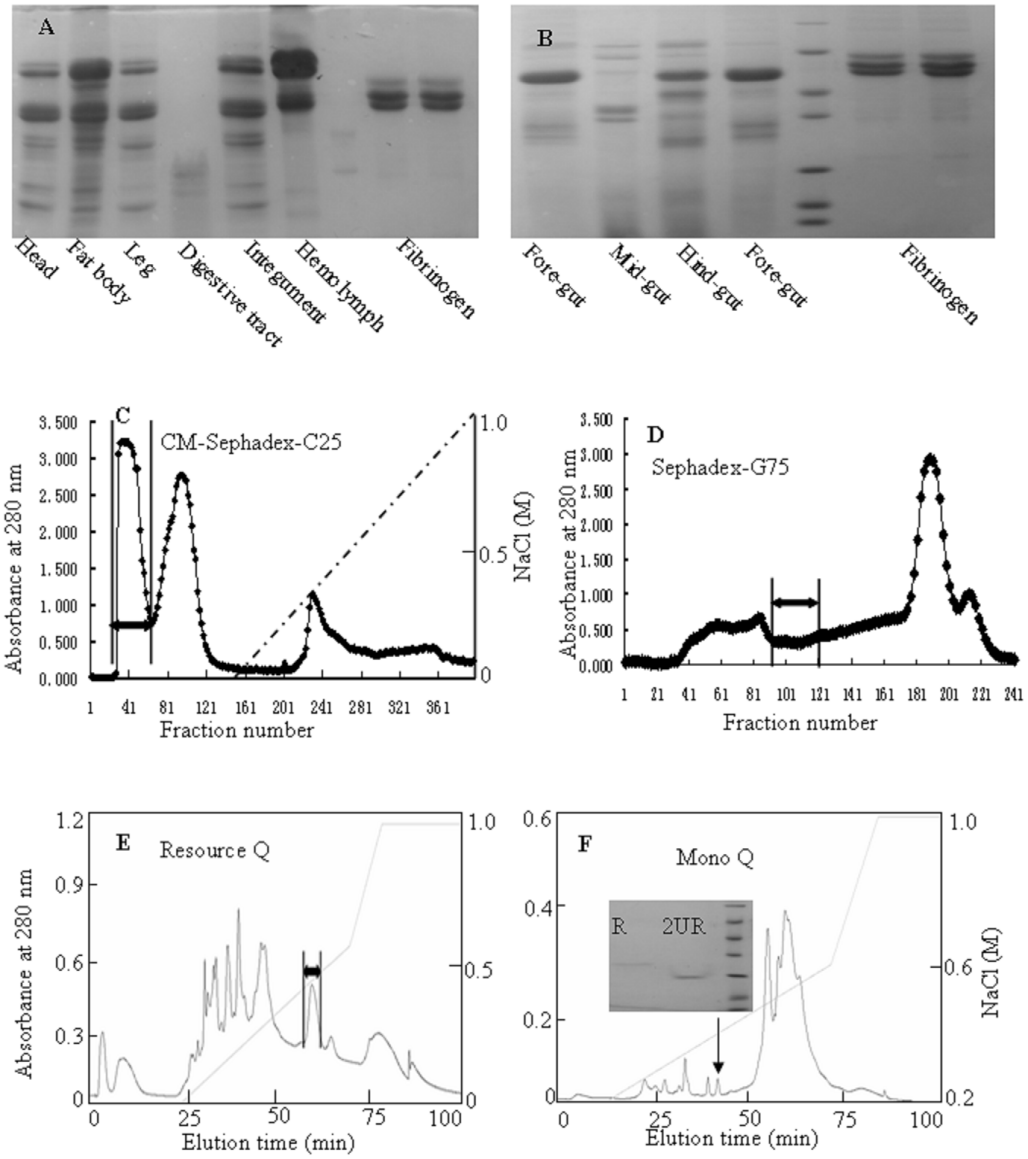
Purification of fibriongenolytic molecules from mid-guts of *E. sinensis*. A and B: tissues extrats from legs, integuments, fat bodies, heads, digestive tracts and hemolymph of *E. sinensis* were used to test their thrombolytic abilities against fibrinogen. 5 µg total extracted protein was incubated with 20 µg fibrinogen for 10 h at 37°C and subjected reduced SDS-PAGE analysis in a gel concentration of 15%. C: cationic exchange chromatography of aliquot of 1.0 g MGS by CM-Sephadex C-25 (Amersham Biosciences, 2.6×40 cm). The interesting peak was indicated by an arrow. D: the collected fraction containing fibrinogenolytic activity from “C” was subjected to Sephadex G-75 gel filtration (Amersham Biosciences, superfine, 2.6×100 cm), the elution was performed by 0.1 M Tris-HCl pH 7.8 with a flow rate of 0.3 ml/min. E: The collected fraction from “D” was subjected to AKTA FPLC Resource Q (1 ml volume, Amersham Biosciences) anionic exchange equilibrated with 0.02 M Tris-HCl pH 7.8. The elution was performed at a flow rate of 1 ml/min with the indicated NaCl gradient. F: the collected fraction from “E” was subjected to AKTA FPLC Mono Q (1 ml volume, Amersham Biosciences) anionic exchange equilibrated with 0.02 M Tris-HCl pH 8.3. The elution was performed at a flow rate of 1 ml/min with the indicated NaCl gradient. The purified protein containing fibrinogenolytic activity was indicated by an arrow. Inset in Fig. 1F: SDS-PAGE analysis of purified protein as indicated in Fig. 1F in 15% gel concentration. R: reduced; UR: Unreduced.

As illustrated in Fig. 1A, all the test tissues or organs (Legs, integuments, fat bodies, heads, digestive tracts and hemolymph) in *E. sinensis* had fibrinogenolytic ability. Compared with other tissues or organs containing activity to only hydrolyze α-chain of fibrinogen, digestive tracts of *E. sinensis* can hydrolyze α-, β-, and λ-chains of fibrinogen. Further analysis indicated that mid-gut could hydrolyze α-, β-, and λ-chains of fibrinogen while fore-gut and hind-gut only hydrolyzed α- and β-chains (Fig. 1B).

### Purification of fibriongenolytic molecules from mid-guts of the beetle

As indicated in Fig. 1C, four protein peaks were eluted from the supernatant of the beetle mid-gut extracts by CM-Sephadex C-25 cationic exchange column. The first peak showed fibrinogenolytic activity and subjected further fractionation by Sephadex G-75 gel filtration (Fig. 1D). The pooled fractions indicated by an arrow in Fig. 1D contained fibrinogenolytic activity was applied to an anionic exchange FPLC column of Resource Q and more than ten protein peaks were eluted out (Fig. 1E). The fibrinogenolytic activity was concentrated on the eluted peak indicated by an arrow in Fig. 1E. The final purification of fibrinogenolytic molecule was performed by an anionic exchange FPLC column of Mono Q (Fig. 1F). A fibrinogenolytic protein named eupolytin1 (indicated by an arrow in Fig. 1F) was purified form the last step. The SDS-PAGE analysis indicated that it is homogenates with molecular weight of 26 kDa (inset in Fig. 1F).

### Identification and characterization of eupolytin1

**Figure 2:**
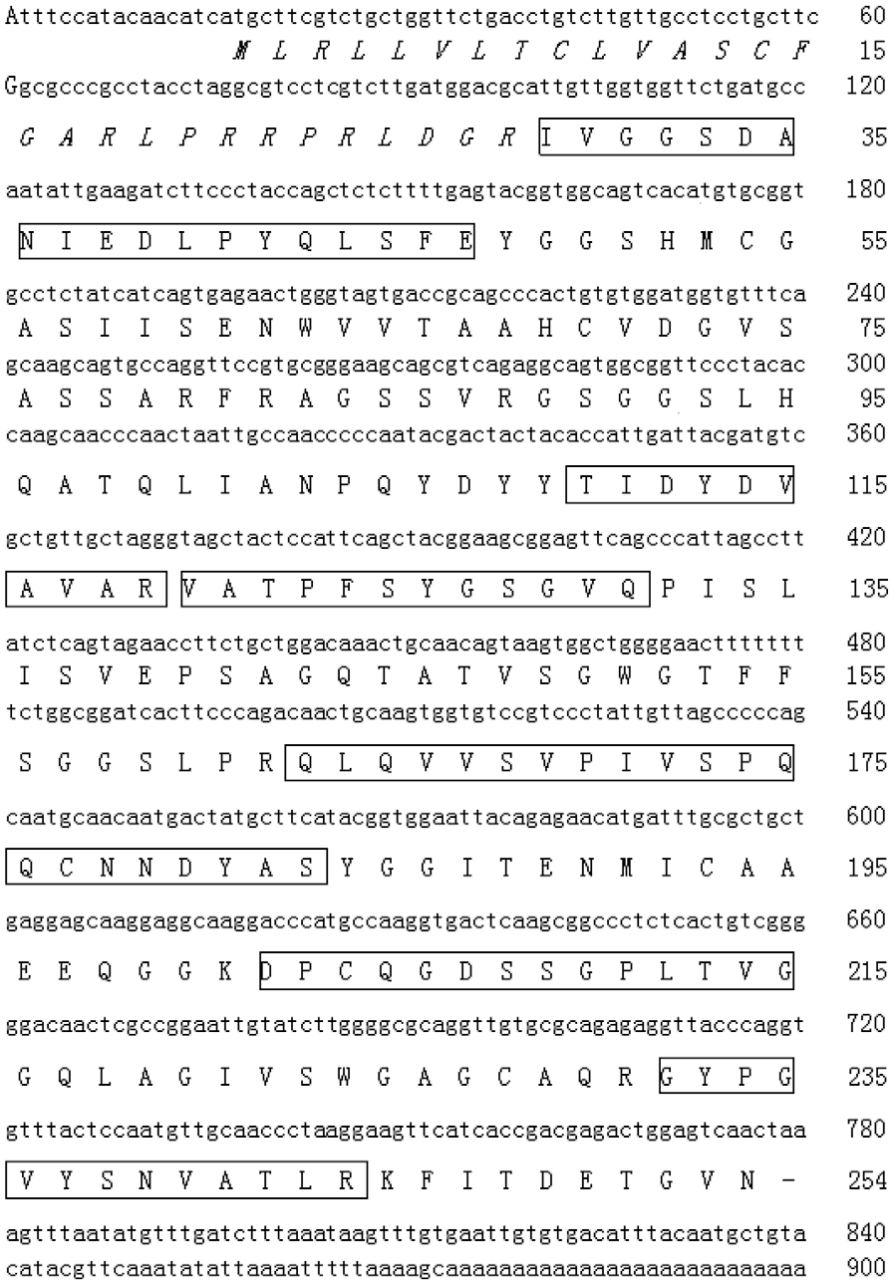
Amino acid sequences of eupolytin1 and its cDNA sequence. The predicted signal peptide is italic. Amino sequences determined by Edman degradation were underlined.

**Figure 3:**
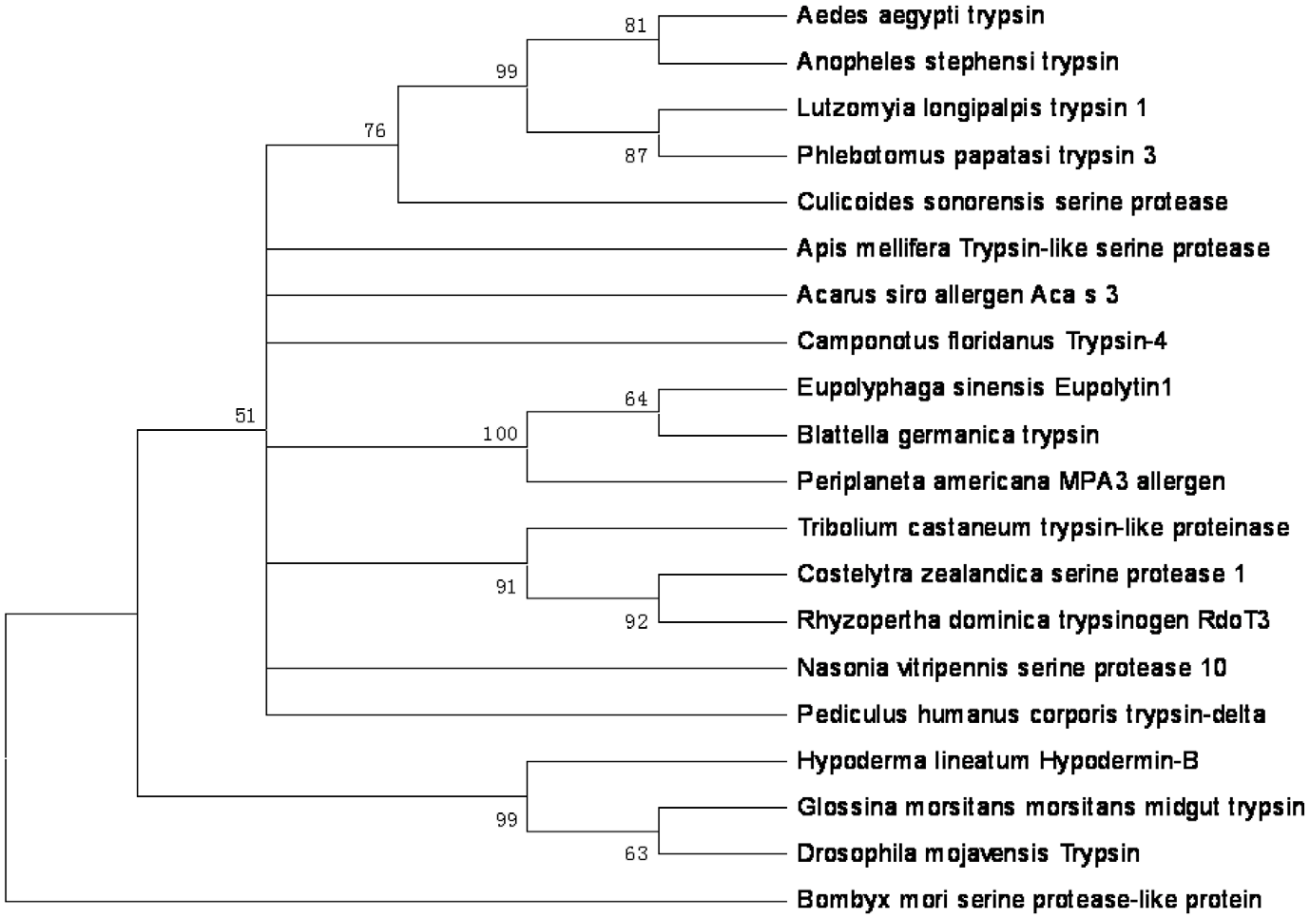
Phylogenetic analyses of eupolytin 1 and other insect serine proteases on basis of the whole precursor sequences. The phylogenetic dendrogram was constructed by the neighbor-joining method based on the proportion difference of aligned amino acid sites of the sequence. Only branches supported by a bootstrap value of at least 50% (expressed as percentage of 500 bootstrap samples supporting the branch are shown at branching points.

A purified fibrinogenolytic protein (eupolytin1) was subjected to amino acid sequence analysis by Edman degradation. The amino acid sequence of the amino-terminal and partial interior amino acid fragments recovered from the trypsin hydrolysis are illustrated in Fig. 2. Based on the amino acid sequences of the N-terminus, degenerated primers were designed to screen the cDNA clones with sequences encoding eupolytin1. The complete cDNA sequences encoding eupolytin1 was cloned from the mit-gut cDNA library of the beetle (GenBank accession Gu187070). The cDNA encodes the proprotein composed of 254 amino acid (aa) residues including predicted signal peptides (27 aa) and mature eupolytin1 (Fig. 2). By BLAST search, it shows 40–50% identity with insect serine proteases. Three conserved amino acid residues (His69, Asp114 and Ser208) are predicted to form catalytic triad for serine proteases. The insect serine protease multi-sequence alignments were performed on basis of the whole sequence of precursor. The condensed multifurcating tree was constructed emphasizing the reliable portion of pattern branches (Fig. 3).

### Fibrinogenolytic activities and effect factors

**Figure 4:**
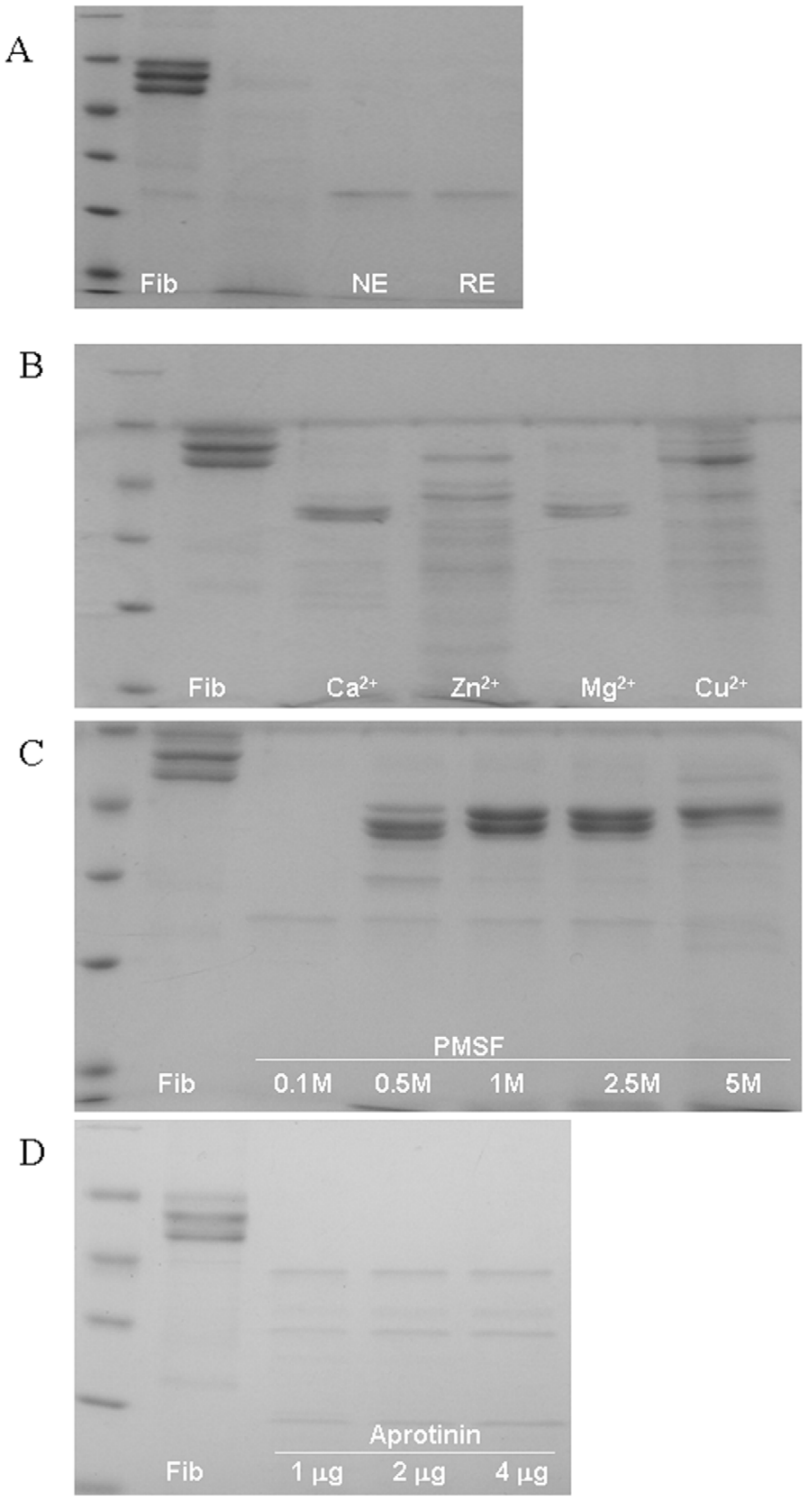
Fibrinogenolytic activity of eupolytin1 and its effects by metal ions and protease inhibitors. A: native and recombinant eupolytin1 (1 µg) were incubated with 20 µg fibrinogen for 1 h at 37°C and subjected reduced SDS-PAGE analysis in a gel concentration of 15%; B: 1 µg eupolytin1 was incubated with 20 µg fibrinogen for 1 h at 37°C in the presence of 5 mM indicated metal ion and subjected reduced SDS-PAGE analysis in a gel concentration of 15%; C & D: 1 µg eupolytin1 was incubated with 20 µg fibrinogen for 1 h at 37°C in the presence of PMSF (C) or aprotinin (D) with indicated concentration and subjected reduced SDS-PAGE analysis in a gel concentration of 15%. NE: native eupolytin1; RE: recombinant eupolytin1.

**Figure 5 pone-0017519-g005:**
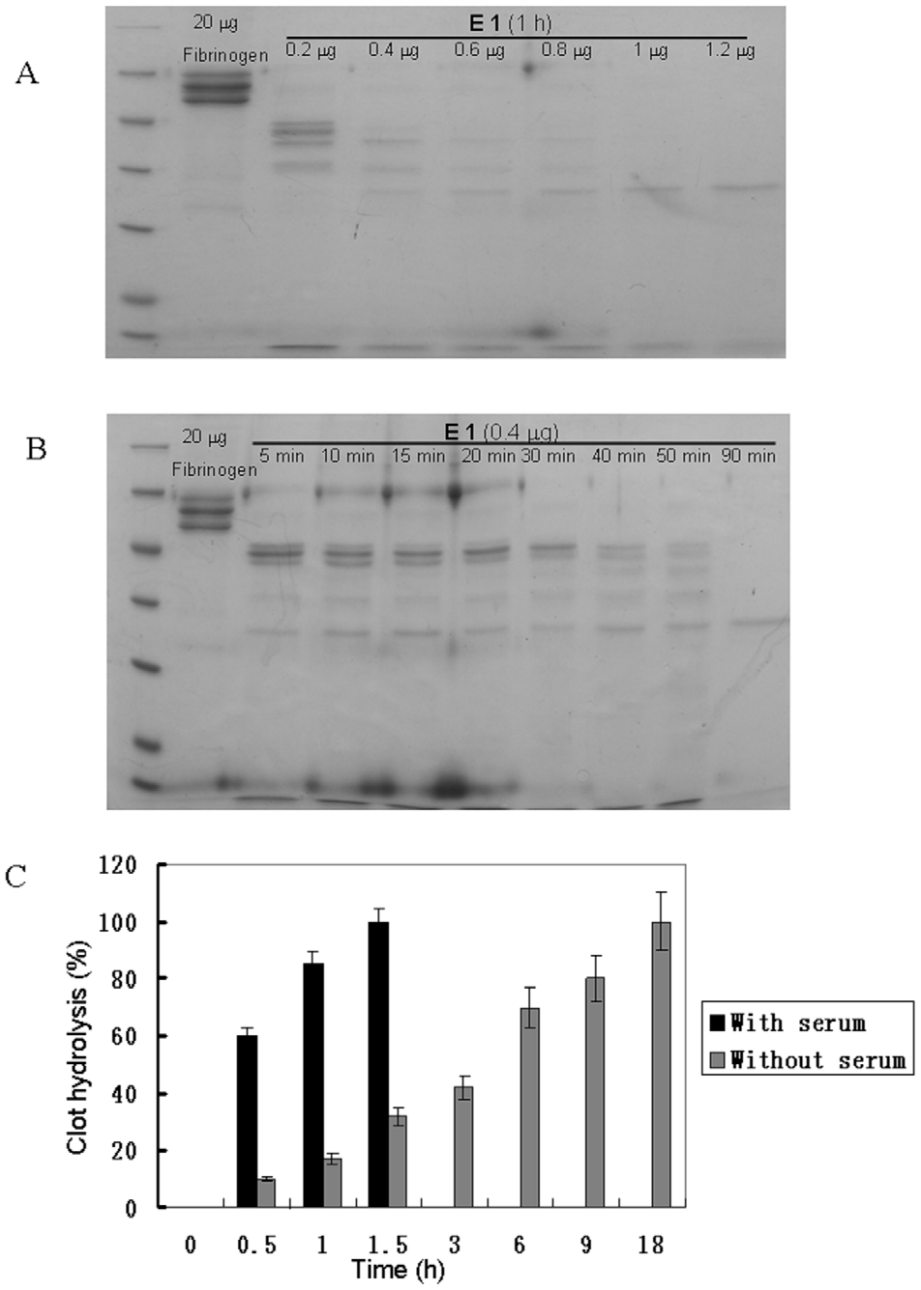
Eupolytin1 hydrolyzed fibrinogen in dose- (A) and time-dependent manners (B). E: eupolytin1.

Native and recombinant eupolytin1 (1 µg) were incubated with 20 µg fibrinogen in 20 µl ABS at 37°C for 1 h and subjected to SDS-PAGE analysis, respectively (Fig. 4A). They showed strong fibrinogenolytic activities. Eupolytin1 could hydrolyze three chains (α, β and λ) of fibrinogen. The effects of metal ions, PMSF and EDTA on eupolytin1 were illustrated in Fig. 4B–C. Ca^2+^ and Mg^2+^ had little effect on fibrinogenolytic activity of eupolytin1. Zn^2+^ and Cu^2+^ appeared to have a little inhibition on fibrinogenolytic activity of eupolytin1. PMSF could significantly inhibit the activity of eupolytin1. EDTA had no effect on eupolytin1 (Data not shown). The result of anti-plasmin-interference experiment was displayed in Fig. 4D. It was observed that the fibrinogen was still hydrolyzed by eupolytin1 with the presence of aprotinin, suggesting that eupolytin1 may exert the fibrin(ogen)olytic activity directly in addition to its plasminogen-activating activity. Fig. 5A and B showed that eupolytin1 hydrolyzed fibrinogen in dose- and time-dependent manners. Eupolytin1 only took 1.5 h to completely hydrolyze 8 mg human whole clot with serum while it took 18 h to completely hydrolyze the clot without serum (Fig. 5C), suggesting that eupolytin1 has an ability to activate plasminogen present in the serum. Kinetic parameters of native and recombinant eupolytin1 for different chromogenic substrates were listed in table 1. They could hydrolyze substrates of T6140 and B3133. T6140 is the special substrate for plasmin while B3133 is the substrate for tyrpsin-like serine proteases. The current results indicated that eupolytin1 had activities of plasmin- and tyrpsin-like serine proteases.

**Table 1:** Kinetic parameters of eupolytin1 for different chromogenic substrates.

	T6140		B3133	
	NE	RE	NE	RE
*Km* (mM)	20.17	20.02	3.24	3.01
*Vmax* (mM.S^−1^)	2.47	2.39	1.05	1.12
*Kcat* (10^5^ S^−1^)	2.10	2.25	0.32	0.29
*Kcat/Km* (10^5^ mM^−1^ S^−1^)	0.10	0.11	0.10	0.08

These data represent mean values of three independent experiments. NE: native eupolytin1; RE: Recombinant eupolytin1.

### Plasminogen activation by eupolytin1

**Figure 6:**
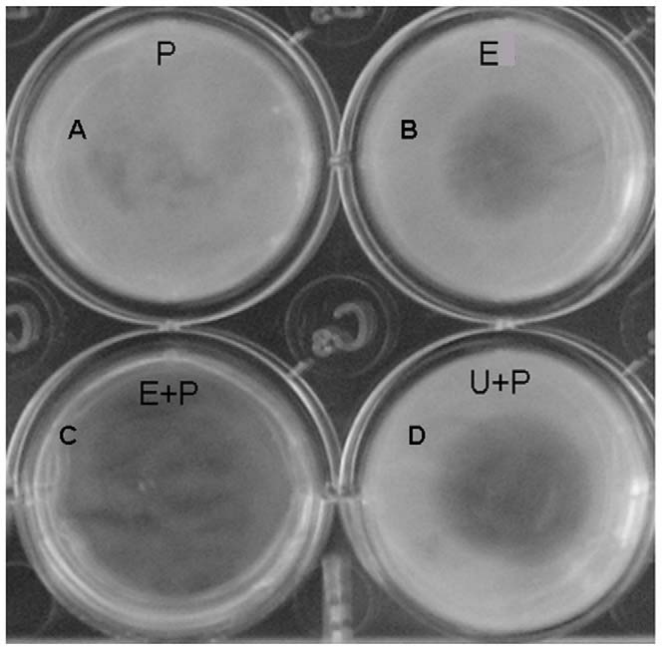
Assays of plasminogen activation and fibrinolysis of eupolytin1. (A) 5 µl plasminogen (11.2 µM); (B) 5 µl eupolytin 1 (3.5 µM); (C) 5 µl eupolytin 1 (3.5 µM) incubated with 5 µl plasminogen (11.2 µM); (D) 5 µl urokinase (3.5 µM) incubated with 5 µl plasminogen (11.2 µM). E: eupolytin1; U: urokinase; P: plasminogen.

Using human urokinase as a positive control, eupolytin1's ability to activate human plasminogen was assayed. Eupolytin1 could activate plasminogen as illustrated in Fig. 6. In the absence of plasminogen, eupolytin1 could hydrolyze fibrin (Fig. 6B); in the presence of plasminogen, its ability to hydrolyze fibrin was increased (Fig. 6A and C). This result indicated that eupolytin1 not only directly hydrolyzed fibrin but also activated plasminogen to hydrolyze fibrin. To the best of our knowledge, this is the first report of the bi-functional proteins containing both fibrinolysis and plasminogen-activation functions.

### *In vivo* anti-thrombosis activity

**Figure 7:**
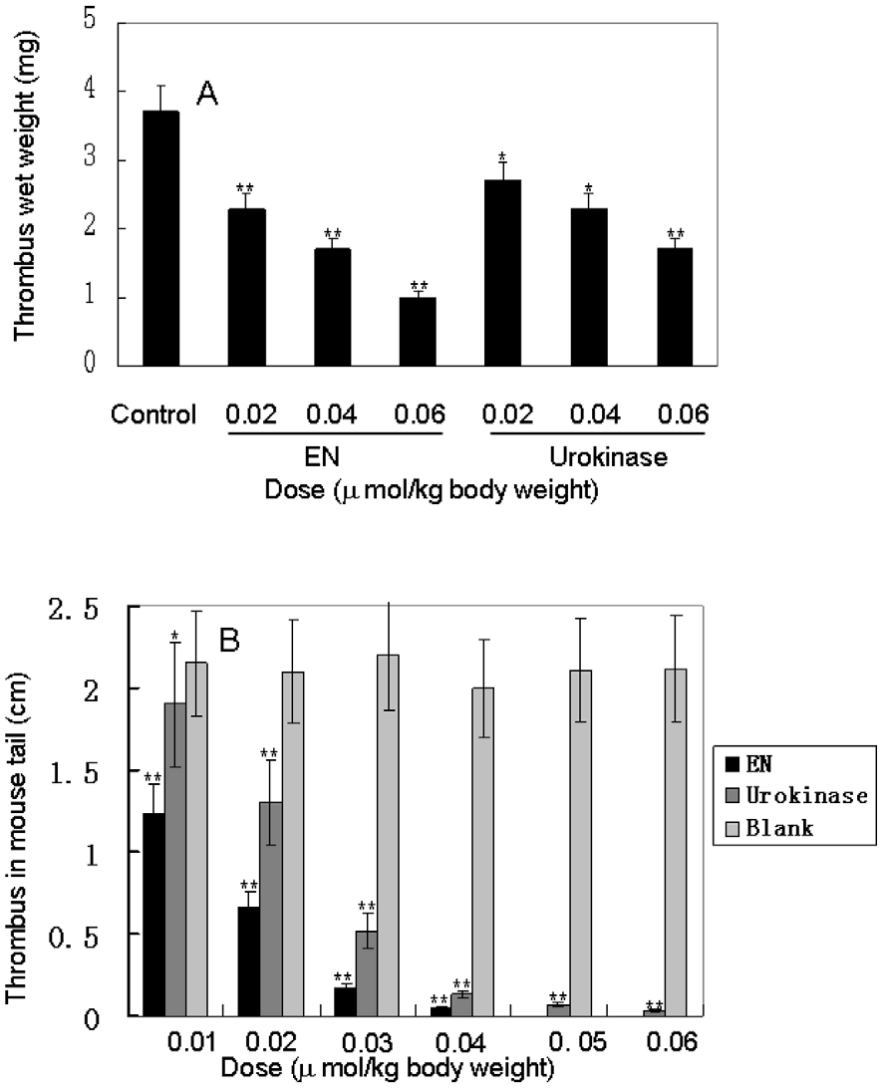
Anti-thrombosis activities of eupolytin1 *in vivo*. A: Effects of eupolytin1 and urokinase on thrombus formation induced by contact activation in rat arteriovenous shunt models; B: Effects of eupolytin1 and urokinase on thrombus formation induced by carrageenan in mouse tail. EN: eupolytin1. * *P*<0.05 and ** *P*<0.01 from saline control.

As illustrated in Fig. 7A, an average weight of 3.7±0.3 mg thrombi were formed in the nylon strings after 5 min of perfusion in the control (saline) arteriovenous shunt rat models. Administration of eupolytin1 and urokinase obviously reduced thrombus weight in a dose-dependent manner. The thrombus weight was reduced to 1±0.2 mg by eupolytin1 administration of 0.06 µmol/kg. At the same dose of urokinase, the thrombus weight was reduced to 1.7±0.3 mg. Thrombosis induced by carrageenin in mice tail could also significantly inhibited by eupolytin1 and urokinase in a dose-dependent manner as illustrated in Fig. 7B. 0.01–0.06-µmol dosage of eupolytin1 could completely scavenge the induced thrombus after 48 h treatment. In all the tested dosages, the anti-thrombosis ability of eupolytin1 was much better than that of urokinase.

### Hemostatic safety

**Figure 8:**
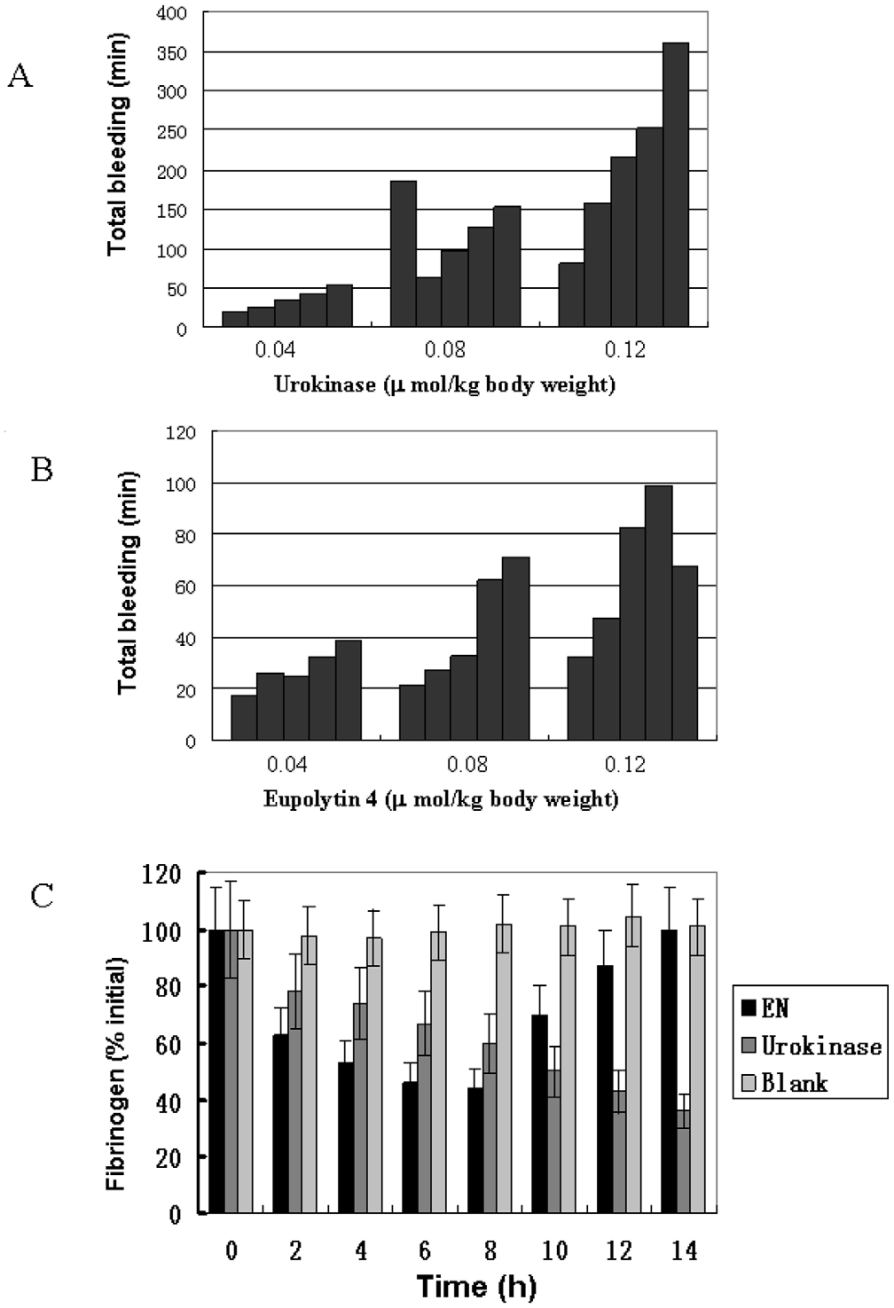
Hemostatic evaluation induced by urokinase (A) and eupolytin1 administration in mice (B). (C) Fibrinogen concentration change induced by urokinase and eupolytin1 administrations in mice. EN: eupolytin1.

Five animals that were administered with saline control showed no change in PBT (2.5±0.7 min). All the tested dosages of eupolytin1 had no significant effects on the PBT. At dosages of 0.08 and 0.12 µmol/kg body weight, urokinase strikingly prolonged the PBT (Table 1). Fig. 8A and B showed the absolute total duration of bleeding for each experimental rabbit. Bleeding for more than 50 minutes occurred at all rabbits receiving urokinase treatment of 0.08 and 0.12 µmol/kg. One of five rabbits receiving urokinase treatment of 0.04 µmol/kg also had total bleeding for more than 50 min. Most of them were more than 150 min; one of them was up to 360 min. Two of five, three of five rabbits receiving eupolytin treatment of 0.08 and 0.12 µmol/kg, respectively, have been found bleeding for more than 50 min but always less than 100 min (Fig. 8A and B). ELISA was used to assess kinetics of fibrinogen level in animal plasma. As illustrated in Fig. 8C, 0.08 µmol-dosage urokinase progressively decreased the concentration of fibrinogen in plasma, and such decline was not stopped yet even after 24 h treatment (Data not shown). A 0.08 µ mol-dosage eupolytin1 also progressively decreased the concentration of fibrinogen in plasma, but this stepwise decrease only lasted 8 h. Afterwards, the plasma fibrinogen concentration gradually went back to normal level (Fig. 8C).

## Discussion

All of the thrombolytics in current clinical use are PAs. One side they have efficacy to dissolve the thrombi, but the other side they may potentially cause the bleeding complications [1]. Lots of efforts have been paid to discover the new thrombolytic agents to improve efficacy of thrombosis but avoid bleeding risk meanwhile. In addition, some position such as cerebrosoinal fluid (CSF) contain little plasminogen, being 1/10 compared with that in the blood [15]–[17]. The lower the concentration of plasminogen surrounding a clot results in the lower the rate of clot lysis. A novel class of thrombolytics, namely, “direct-acting” agents, which have ability to directly dissolve thrombi and need no activation of plasminogen, was considered to have ability for achieving safe thrombolytic therapy. One of “direct-acting” agents is plasmin. However, plasma inhibitor neutralizes intravenously-administered plasmin [1]–[3]. Exogenous “direct-acting” agents containing resistance against plasma inhibitor may be excellent thrombolytic candidates.

A thrombolytic protein (Eupolytin1) was purified and characterized from mid-guts of the medicinal insect of *E. sinensi*. Native eupolytin1 is composed of 227 aa. BLAST search revealed that it is a novel protein belonging to serine protease family. Native and recombinant eupolytin1 could hydrolyze chromogenic substrates T6140 and B3133 (Table 1), indicating that eupolytin1 contains plasmin- and trypsin-like activities. Similar to plasmin, eupolytin1 contains ability to hydrolyze fibrinogen and fibrin. Native and recombinant eupolytin1 showed strong fibrino(gen)olytic activities; 20 µg fibrinogen could be completely hydrolyzed by 1 µg eupolytin1 during 1.5 h (Fig. 4 and 5). Different from known fibrino(gen)olytic enzymes which only hydrolyze α- or/and β-chains of fibrinogen [8], [9], eupolytin1 could hydrolyze α-, β-, and γ-chains of fibrinogen. The current fibrino(gen)olytic enzyme combined with its strong fibrino(gen)olytic activity revealed the anti-thrombosis mechanism of the traditional medicinal insect, *E. sinensi*.

As mentioned above, eupolytin1 had ability to activate human plasminogen as tPA or uPA did. Fig. 4–6 showed that in addition to directly hydrolyzing fibrin(ogen) (direct-acting) (Fig. 4, Fig. 5, Fig. 6B), eupolytin1 could activate plasminogen to hydrolyze fibrin (indirect-acting) (Fig. 6A and C). As illustrated in Fig. 6A and C, the synergetic action of these two means (direct-acting and indirect-acting) obviously improved the fibrinolysis efficiency of eupolytin1. Such type of bi-functional agent containing both plasmin- and PA-like activities has more advantages than known anti-thrombosis agents to treat thrombus.

As mentioned above, eupolytin1 not only directly hydrolyzes thrombus as plasmin does, but also activates intrinsic plasminogen to exert anti-thrombosis functions as PA dose. Based on the unique bi-functional characteristic, we assumed that eupolytin1 has excellent anti-thrombosis ability *in vivo* and *in vitro*. The experiments of *in vitro* clot lysis with human blood indicated that the clot lysis rate of eupolytin1 with serum (1. 5 h) is much faster than that of eupolytin1 without serum (18 h) (Fig. 5C). This result indicated that eupolytin1 possibly activated plasminogen in the serum to hydrolyze the blood clot. Using urokinase as a control, the thrombolytic results *in vivo* further confirmed that eupolytin1 has excellent anti-thrombosis ability (Fig. 7). In the both *in vivo* anti-thrombosis models (arteriovenous shunt rat model and thrombosis induced by carrageenin in mice tail), eupolytin1 showed much better anti-thrombosis effects than urokinase.

The risk of hemorrhage was evaluated after eupolytin1 administration in rabbit (Table 2, Fig. 8A and B). Compared with urokinase, eupolytin1 induced little bleeding even the dose up to 0.12 µmol/kg body weight. This dose was three times of the tested dose that could completely lyse experimental thrombi in mice (Fig. 7). In order to reveal the reason to induce little bleeding by eupolytin1, the fibrinogen concentration change in rabbit plasma was traced as illustrated in Fig. 8C. Eupolytin1 could rapidly decrease plasma fibrinogen concentration. The lowest fibrinogen concentration could be reached after 8 h eupolytin1 administration, and then returned to normal level after 14 h. The unique kinetics characteristic of plasma fibrinogen concentration induced by eupolytin1 not only increases its rate of clot lysis but also decreases its hemorrhage risk.

**Table 2:** Mean ± SD of primary bleeding time relative to start of eupolytin1 or urokinase infusion.

	Eupolytin1 (µmol/kg)			Urokinase (µmol/kg)		
	0.04	0.08	0.12	0.04	0.08	0.12
Before treatment	2.3±0.3	2.5±0.6	2.2±0.7	2.6±0.8	2.3±0.3	2.7±0.2
Treatment	2.8±0.9	2.2±0.5	2.9±1.3	2.9±0.6	6.5±4.3	10.7±4.7
After treatment	2.9±1.0	3.2±0.7	3.6±2.9	2.3±0.2	7.3±6.4	12.9±5.8

Preatment times were −30, −10, and 0 minutes; treatment times, 10 and 30 minutes; and posttreatment times, 60, 90, 120 and 180 minutes.

Further work is necessary to study the structure-function relationship of the thrombolytic reagent and evaluate its possibility to be used as safe clinical thrombolytics. In addition, eupolytin1 has a possibility to acts as “direct-acting” thrombolytic agents in some special position such as CSF containing little plasminogen.

## Materials and Methods

### Insects

Thirty kg *E. sinensis* (about 75 000, average weight 0. 4 g) was reared in the laboratory with free accesses to foods (corn, fish and vegetable) and water. The laboratory temperature is 23±2°C. All the animal experiments are approved by Kunming Institute of Zoology, Chinese Academy of Sciences.

### The determination of anatomical position with fibrinogenolytic activity

Legs, integuments, fat bodies, heads, digestive tracts and hemolymph of *E. sinensis* were dissected and collected, respectively. These isolated organs or tissues were homogenized in 0.05 M acetate-acetate sodium buffer solution, pH 5.0 (ABS), and centrifuged at 5000 *g* for 10 min. The supernatants were used to test their thrombolytic abilities against fibrinogen as described below.

### Purification of thrombolytic component from the mid-guts of *E. sinensis*

40 g mid-gut collected from ten kg *E. sinensis* was homogenized in 500 ml 0.05 M ABS, and centrifuged at 5000 g for 10 min. The mid-gut supernatants (MGS) were lyophilized (80 g, containing salt) and kept at −80°C. Aliquot of 1.0 g MGS (totaling 80 aliquots) dissolved in 10 ml 0.05 M ABS was dialyzed against 3 L 0.05 M ABS for 12 h, and then was applied to a CM-Sephadex C-25 (Amersham Biosciences, 2.6×40 cm) equilibrated with 0.05 M ABS. The elution was performed at a flow rate of 1 ml/min with the indicated NaCl gradient in 0.05 M ABS, with fractions collected every 3.0 ml. The absorbance of the eluate was monitored at 280 nm (Fig. 1C). Every fraction was subjected to pharmacological testing including inhibition of platelet aggregation, serine protease hydrolysis on chromogenic substrates, blood coagulation and fibrinogenolysis as indicated in the experimental protocol. Protein peaks containing tested pharmacological activities were pooled and purified further by Sephadex G-75 gel filtration, Resource Q and Mono Q anionic exchange fast protein liquid chromatography (FPLC) columns as illustrated in Fig. 1D–F. All the purified interesting proteins from 80 aliquots of MGS were pooled and subjected to further study.

### Structural analysis

Amino acid sequences of the N-terminus and partial interior peptide fragments recovered from trypsin hydrolysis were determined by automated Edman degradation on an Applied Biosystems pulsed liquid-phase sequencer, model 491.

### SDS-Polyacrylamide Gel Electrophoresis (SDS-PAGE) analysis and protein concentration determination

SDS-PAGE was performed under reduced and/or un-reduced conditions. Protein samples were loaded onto a 15% polyacrylamide gel. Protein bands were observed after using a standard Comassie blue stain. The protein concentration was determined by a protein assay kit (Bio-Rad, Hercules, CA) with bovine serum albumin as a standard.

### cDNA library construction,cDNA screening and phylogenetic tree construction

Total RNA was extracted using TRIzol (Life Technologies, Ltd.) from 60 mid-guts of *E. sinensis*. cDNA was synthesized by SMART™ techniques by using a SMART™ PCR cDNA synthesis kit (Clontech, Palo Alto, CA) according to the manufacturer instructions. A directional cDNA library was constructed with a plasmid cloning kit (SuperScript™ Plasmid System, GIBCO/BRL) following the instructions of manufacturer, producing a library of about 5.7×10^5^ independent colonies. PCR-based method for high stringency screening of DNA libraries was used for screening and isolating clones. The specific primers in the sense direction as listed in Table S1 designed according to the peptide fragment sequences of determined by Edman degradation and primer IIA (5′-AAGCAGTGGTATCAACGCAGAGT-3′) provided by the SMART™ PCR cDNA synthesis kit in the antisense direction were used in PCR reactions. The DNA polymerase was Advantage polymerase from Clontech (Palo Alto, CA). The PCR conditions were: 2 min at 94°C, followed by 30 cycles of 10 sec at 92°C, 30 sec at 53°C, 40 sec at 72°C. DNA sequencing was performed on an Applied Biosystems DNA sequencer, model ABI PRISM 377.

Totally twenty insect serine protease sequences were obtained from the protein database at the National Center for Biotechnology Information. Multiple sequence alignments were constructed by using the ClustalW program (version 1.8) on basis of the whole recursor of these serine proteases. The phylogenetic trees were constructed using neighbor-joining method (MEGA4.0), by calculating the proportion of amino acid differences (p-distance) among all sequences. A total of 500 bootstrap replicates were used to test the reliability of each branch. The numbers on the branches indicate the percentage of 500 bootstrap samples supporting the branch.

### Recombinant expression of eupolytin1

The coding region including restriction enzyme sites for eupolytin1 was isolated from the cDNA library. Two PCRs were performed to insert enzymatic processing sites for enterokinase, HindIII and KPN1 The primer pair for the first PCR to introduce enzymatic processing sites for enterokinase and HindIII is 5′-BD1810-1: 5′-GATGATGACAAGATTGTTGGAGGTT-3′ and 3′-BD1810-1: 5′-CCCCCAAGCTTCTAGTTGACTCCAG-3′. The primer pair for the second PCR to introduce enzymatic processing site for KPN1 is 5′-BD1810-2: 5′-CGGGGTACCGATGATGATGACAAG-3′ and 3′-BD1810-1. PCRs were performed by running 30 cycles with a temperature profile of 30 s at 95°C, 30 s at 57°C, and 60 s at 72°C. The purified PCR product was digested with HindIII and KPN1, and ligated into the pET-32a (+) plasmid at the corresponding restriction sites. Expression, purification and refolding of the recombinant His-tagged protein were performed according to the manufacturer's instruction (Novagen). Recombinant protein was released by enterokinase from the fusion protein. It was purified from the digestive mixture by AKTA FPLC Mono Q anionic exchange column as mentioned above (Fig. S1).

### Serine protease and fibrinogenolytic assays

Serine protease and fibrinogenolytic assays of the native purified protein were carried out according to our previous method [8], [9]. The hydrolysis of synthetic chromogenic substrates by the tested samples was assayed in 50 mM Tris–HCl, pH 7.8 at 37°C. The substrates are T6140 (N-(p-Tosyl)-Gly-Pro-Lys 4-nitroanilide acetate salt, Sigma) and B-3133 (N-Benzoyl-Arg-4-nitroanilide-hydrochride-pNA, Sigma). The reaction was initiated by the addition of the substrate to a final concentration of 0.5 mM. The formation of p-nitroaniline was monitored continuously at 405 nm for 5 min. 1 µl 20 mg/ml bovine fibrinogen was incubated with the tested samples in 25 µl 50 mM Tris-HCl, pH 7.6 containing 150 mM NaCl at 37°C for different times, and then subjected to reduced SDS-PAGE analysis on a gel with a concentration of 12%. The inhibition on platelet aggregation was determined according to previous method [9]. Platelet aggregation was monitored by light transmission in an aggregometer (Plisen, Beijing) with continuous stirring at 37°C.

### *In vitro* clot lysis with human blood

Clots were formed by adding thrombin and excess calcium into health human whole blood. After 90 min incubation at 37°C, the retracted clots were packed into 5-cm sections. Test samples (native protein) were administered into the clots and the lysis was visually monitored. All the human experiments are approved by Kunming Institute of Zoology, Chinese Academy of Sciences. The informed consent from all participants involved in your study was obtained in written, and the consent procedure was also approved by Kunming Institute of Zoology, Chinese Academy of Sciences.

### Plasminogen activation and fibrinolysis assay

A fibrin plate lysis assay was conducted in a 12-well plate. A mixture consisting of 1 ml of fibrinogen (5 mg/ml), 1 ml of 1% agarose solution and thrombin (1 unit) in 20 mM Tris–HCl buffer, pH 7.6, containing 0.1 M NaCl was poured into each well. After 1 h incubation at room temperature, the native sample was placed on the surface and incubated overnight at 37°C. The diameter of the lysed areas was measured. Plasminogen (with no plasmin) and urokinase were used as negative and positive control, respectively. In the fibrinolysis assay, the drug aprotinin (Sigma, A4529, 1, 2, 4 µg), which inhibits trypsin and related proteolytic enzymes, was mixed with 1 µg eupolytin1 sample, and incubated with 20 µg fibrinogen in order to eliminate the interference of plasmin, which would be produced by eupolytin1 activation if there was plasminogen contaminated in the sample.

### Anti-thrombosis study by rat arteriovenous shunt thrombosis model

Male Wistar rats (180–220 g) were used for rat arteriovenous shunt thrombosis model according the method described by Sperzel and Huetter [18]. Rats were anesthetized by intraperitoneal (i.p.) administration of ketamine (50 mg/kg) and xylazine (15 mg kg). The left jugular vein and right common carotid artery were isolated and catheterized by a shunt catheter (American Health & Medical Supply International Corp. Co., Ltd). This catheter is composed of three parts including two 100-mm-long polyethylene (PE)-60 catheters, which were introduced into the blood vessels, and a 30-mm-long PE-160 catheter that is in the middle of the two PE-60 catheters. A rough nylon thread (60 mm in length and 0.26 mm in diameter) folded into a double string was in the middle PE-160 catheter to induce thrombosis. For sample administration, a saline-filled PE-60 catheter was cannulated to the exposed femoral vein. Different dose of samples (n = 9 per dose) were administered through the PE-60 catheter cannulated to the femoral vein. After 15 min administration of sample, the shunt was opened for 5 min, and then closed. Nylon strings were removed from the middle PE-160 catheter; the wet thrombi attached in the nylon strings were weighed. All the experimental protocols of animal models were approved by the Animal Care and Use Committee at Kunming Institute of Zoology, Chinese Academy of Sciences. The approval ID for this study was syxk2009-0007.

### Anti-thrombosis study by carrageenan-induced mouse tail thrombosis model

Carrageenan-induced mouse tail thrombosis model was used to evaluate antithrombosis *in vivo*. The general procedures were the same as those in previous reports [19]. The tested sample was injected into mice by caudal vein. After 30 min of the injection, 1% κ-Carrageenan dissolved 0.9% NaCl was intraperitoneally injected into mice to induce thrombosis in tails. The tested sample was injected into mice by caudal vein again after 6 h of the carrageenin injection. The length of thrombus in tails of mice was measured after 48 h treatment.

### Fibrinolytic hemorrhage assay *in vivo*

According to previous reports, rabbit ear-puncture technique was used to assess fibrinolytic hemorrhage [20]–[22]. Primary bleeding times (PBT) were performed before, during and after agent infusion, and all lesions were monitored for re-bleeding. Blood samples were collected on citrate anticoagulant (0.5 ml, 0.25 M sodium citrate). After centrifugation at 3000 g at 4°C for 30 min to obtain platelet-poor plasma and tested immediately for fibrinogen concentration.

All the experimental protocols of animal models were approved by the Animal Care and Use Committee at Kunming Institute of Zoology, Chinese Academy of Sciences.

### Statistics

Data were analyzed by *X* ^2^ and by *t* test or repeated measure analysis of variance (ANOVA) comparison of means.

## Supporting Information

Figure S1 **SDS-PAGE of fractions from the purification of recombinant eupolytin1.** Lane 1: un-induced cells. Lane 2: induced cell. Lane 3:purified fusion protein after affinity chromatography. Lane 4: purified protein after enterkinase cleavage. Lane 5: purified protein after Mono-Q FPLC. Lane 6: protein marker.
(TIF)

Table S1 **Primers for cDNA clone screening of eupolytin 1.**
(DOC)
