# Supplementary figures and images for: A Bi-Functional Anti-Thrombosis Protein Containing Both Direct-Acting Fibrin(ogen)olytic and Plasminogen-Activating Activities
Source: PLoS One. 2011 Mar 14;6(3):e17519. doi: 10.1371/journal.pone.0017519 (PMC3056663; doi:10.1371/journal.pone.0017519)

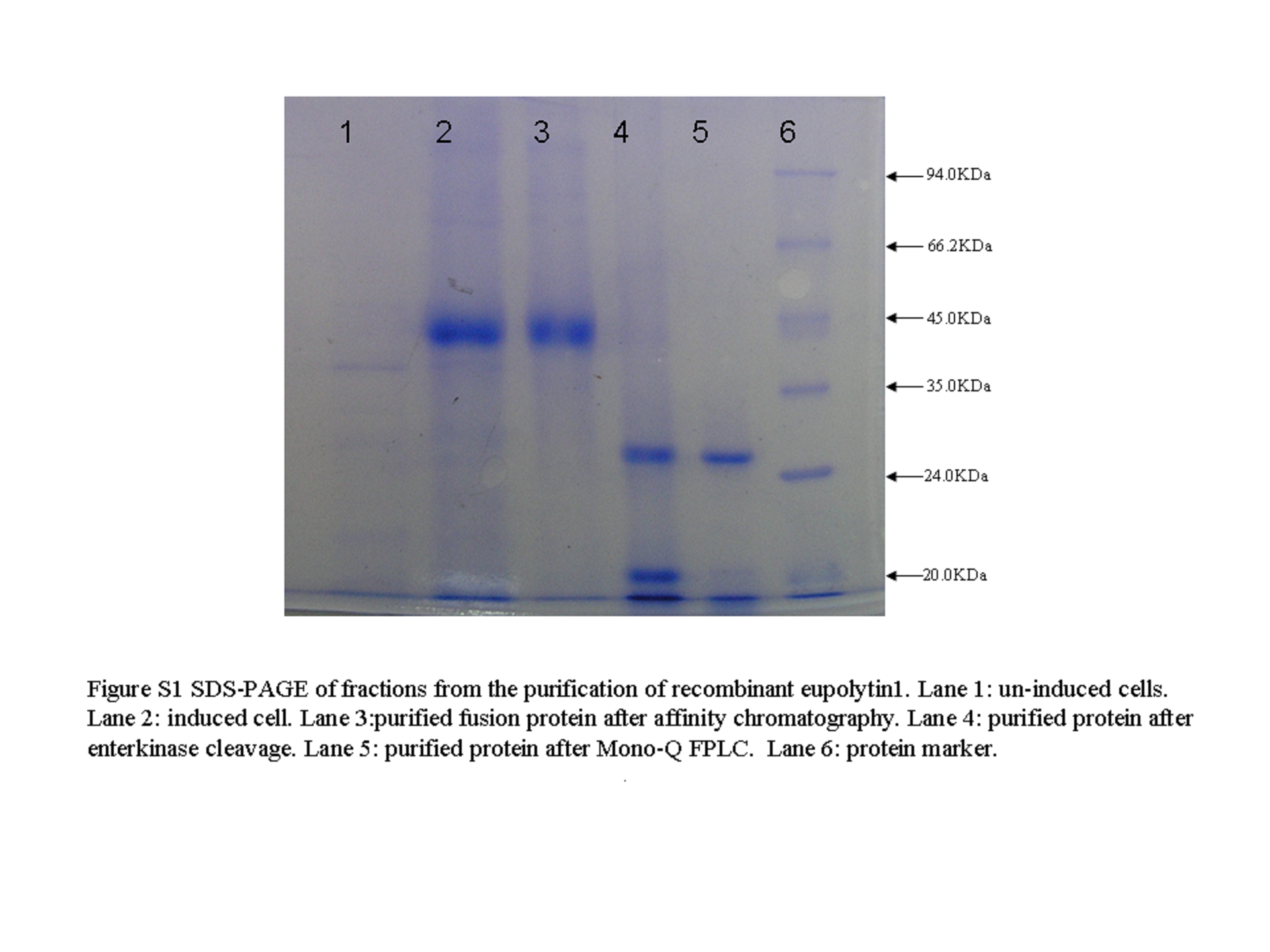

Supplement: Figure S1 — SDS-PAGE of fractions from the purification of recombinant eupolytin1. Lane 1: un-induced cells. Lane 2: induced cell. Lane 3:purified fusion protein after affinity chromatography. Lane 4: purified protein after enterkinase cleavage. Lane 5: purified protein after Mono-Q FPLC. Lane 6: protein marker. (TIF) [file pone.0017519.s001.tif]
