# Supplementary material for: A Bi-Functional Anti-Thrombosis Protein Containing Both Direct-Acting Fibrin(ogen)olytic and Plasminogen-Activating Activities
Source: PLoS One. 2011 Mar 14;6(3):e17519. doi: 10.1371/journal.pone.0017519 (PMC3056663; doi:10.1371/journal.pone.0017519)
Supplement: Table S1 — Primers for cDNA clone screening of eupolytin 1. (DOC) [file pone.0017519.s002.doc]

**Table S1.** Primers for cDNA clone screening of eupolytin 1.

| Primer 1 | AT(A/C/T)GT(A/T/C/G)GG(A/T/C/G)GG(A/T/C/G)AG(T/C)GA(A/T/C)GC(A/T/C/G)AA(A/C/T) |
| --- | --- |
| Primer 2 | AT(A/C/T)GT(A/T/C/G)GG(A/T/C/G)GG(A/T/C/G)AG(T/C) AA(A/C/T)GC(A/T/C/G)TA(C/T) |
